# Synchronous Biodegradability and Production of Dissolved Organic Matter in Two Streams of Varying Land Use

**DOI:** 10.3389/fmicb.2020.568629

**Published:** 2020-11-16

**Authors:** Meredith Kadjeski, Christina Fasching, Marguerite A. Xenopoulos

**Affiliations:** ^1^Environmental and Life Sciences Graduate Program, Trent University, Peterborough, ON, Canada; ^2^Department of Biology, Trent University, Peterborough, ON, Canada

**Keywords:** dissolved organic matter, streams, biodegradability, DOM composition, seasonality, DOC production

## Abstract

In aquatic ecosystems, dissolved organic matter (DOM) composition is driven by land use, microbial activity, and seasonal variation in hydrology and water temperature, and, in turn, its microbial bioavailability is expected to vary due to differences in its composition. It is commonly assumed that DOM of terrestrial origin is resistant to microbial activity because it is composed of more complex aromatic compounds. However, the effect of DOM sources on the microbial reworking and degradation of the DOM pool remains debated. We performed laboratory incubation experiments to examine how temporal changes in DOM composition influence its microbial biodegradability in two contrasting streams (agricultural and forested) in southern Ontario, Canada. Despite a more allochthonous-like DOM signature in the forest stream and a more autochthonous-like DOM signature in the agriculture stream, we found that biodegradation and production of DOC were the same in both streams and synchronous throughout the sampling period. However, the initial DOM composition impacted how the DOM pool changed upon degradation. During the incubations, both autochthonous-like and allochthonous-like fractions of the DOM pool increased. We also found that a greater change in DOM composition during the incubations induced higher degradation of carbon. Finally, temporal variation in DOC biodegradation and production over time or across streams was not related to DOM composition, although there was a significant relationship between BDOC and nutrient concentrations in the agriculture stream. This observation potentially challenges the notion that DOM origin predicts its bioavailability and suggests that broad environmental factors shape DOC consumption and production in aquatic ecosystems. More research is needed to better understand the drivers of microbial biodegradability in streams, as this ultimately determines the fate of DOM in aquatic ecosystems.

## Introduction

In most aquatic systems, dissolved organic matter (DOM) is the largest and most bioavailable form of carbon (Battin et al., 2008) and plays an important role in a variety of environmental and ecological processes. In freshwater ecosystems, DOM composition is mainly related to its origin and can broadly be separated into whether it is derived from terrestrial (allochthonous) or in-stream (autochthonous) primary production. The microbial bioavailability of DOM from these sources differs from one another and varies seasonally. Bioavailable dissolved organic carbon (BDOC) refers to the proportion of DOC removed by bacteria during a fixed period of time. BDOC is most commonly determined via bioassay incubation experiments (in the dark) where changes in DOC concentration are measured over time. The bioavailability of DOC is primarily determined by its chemical composition (Sun et al., 1997; Benner and Opsahl, 2001; Fellman et al., 2009; Williams et al., 2010); specifically, the molecular size of DOM has implications for the bacterial utilization of the DOM (Amon and Benner, 1996; Berggren et al., 2010). Autochthonous-like DOM is dominated by lower molecular weight compounds and is typically considered to be highly degradable (Barrón et al., 2012). By contrast, DOM derived from terrestrial sources is mostly characterized by a complex structure commonly assumed to be less degradable (Kaplan and Bott, 1989; Tranvik, 1992; Jaffé et al., 2008). However, recent studies have found opposing evidence that terrestrial carbon is utilized by aquatic microbial communities (McCallister and del Giorgio, 2012; Fasching et al., 2014; Marín-Spiotta et al., 2014; Stadler et al., 2020). In addition, some studies detect production of DOM during similar incubation experiments (Kaiser and Benner, 2008; Guillemette and del Giorgio, 2012).

Agricultural and urban land use are important watershed drivers controlling the amount, composition, and fate of DOM in aquatic ecosystems (Wilson and Xenopoulos, 2008, 2009; Williams et al., 2010, 2013, 2016; Shang et al., 2018). The composition of DOM in aquatic systems varies based on the different land uses in the surrounding catchments: DOM originating from forested areas is typically dominated by high molecular weight aromatic, humic-like compounds leached from soil and terrestrial litter (Giling et al., 2013; Hulatt et al., 2014). Agricultural catchments on the other hand are sources of lower molecular weight compounds that resemble more protein-like material. These differences in molecular weight and composition may affect in-stream DOM processing. Increases in the abundance of bioavailable autochthonous-like DOM may stimulate microbial activity, as lower molecular weight compounds are generally more labile to microbial consumers and thus highly degradable (Williams et al., 2010; Giling et al., 2013). Variation in DOM composition between watersheds is important because it may determine the bioavailability of the DOM pool that is found within or exported from rivers (Coble et al., 2016).

Streams are not simply a conduit from soils to oceans; within-system processes can also alter the composition of DOM and potentially its bioavailability (Cole et al., 2007; Wiegner et al., 2009; Coble et al., 2016). Processing and transport along the hydrological path alters the composition of DOM through microbial processing, photodegradation, and autochthonous production, and these changes affect the degradation of DOM further downstream (Kaplan and Bott, 1982; Larson et al., 2007; Coble et al., 2016; Fasching et al., 2019). Transformations both within and outside freshwater systems can differ largely and thus may have different consequences for DOM composition and bioavailability (Stutter and Cains, 2016). Therefore, the origin of DOM may be largely responsible for its bioavailability (Wiegner and Seitzinger, 2001; Seitzinger et al., 2002; Guillemette and del Giorgio, 2011) and consequently affect its rate of in-stream processing (Fasching et al., 2014; Berggren and del Giorgio, 2015). In addition, the influences of catchment land use are usually confounded with those associated with other environmental and hydrologic drivers (e.g., water temperature and precipitation) (Wilson et al., 2016).

The interaction of various DOM sources and in-stream microbial processing with temporal patterns—in relation to DOM biodegradability—remains widely debated. In particular, whether BDOC exhibits synchronous behavior across catchments through time or is driven primarily by DOM sources has not yet been examined. Hence, it remains unclear whether catchment land use (i.e., DOM composition) plays a primary role in regulating variability in DOM degradability in aquatic ecosystems and whether the effects of land use are consistent across a range of temporal scales. This study focused on how microbial biodegradability in two contrasting streams with different DOM composition and nutrient concentrations changed over time. To address this, we quantified changes in DOM quantity, composition, and biodegradability in two catchments dominated by different land uses (agricultural and forested) where rainfall and flow are strongly seasonal. We tested the hypothesis that DOM biodegradability is mediated by its composition. Thus, we expected the DOM composition and the associated microbial reworking to differ between the two streams and throughout the year, where the agriculture stream incubations in summer would shift the DOM pool to a more labile composition and enhance its microbial biodegradability.

## Materials and Methods

### Study Area

To assess the impact of landscape character and hydrology on the relative amount of bioavailable dissolved organic carbon (BDOC;% of initial DOC) and the composition of this flux, two contrasting catchments in southern Ontario, Canada, were selected for this study: agricultural (Penville Creek; 44.58751, −79.6655) and forested (Coldwater Creek; 44.13008, −79.7274). The current land use distribution of the agriculture watershed is notably: 47.8% total cropland, 38.1% rural land use, 8.3% forested (wooded and plantation), 0.3% wetland, and 5.5% other/unclassified; while the current land use distribution for the forest watershed is notably: 58.2% forested (wooded and plantation), 23.1% wetland, 2.2% rural land use, 1.5% total cropland, and 15% other/unclassified (Williams et al., 2016). The watershed area differs between the two streams, with the agriculture stream (59.087 km^2^) draining more than twice as much area as the forest stream (22.798 km^2^; Ontario Ministry of Natural Resources and Forestry, 2019). At both sites, monthly air temperature in the warmest months (July and August) is >20°C, and the coldest months (December and January) is between −9 and −7°C (Environment and Climate Change Canada, 2018). Average precipitation in 2018 in the agriculture and forest watersheds was 90.21 and 88.84 cm, respectively (Environment and Climate Change Canada, 2018).

### Field Analyses and Sample Collection

We collected water samples 24 times for each stream between April and November 2018. Samples were collected just below the surface from the same spot in each stream, generally once a week or every 2 weeks, in clean, sample rinsed plastic bottles (soaked in 0.1 M HCl, rinsed with DI water) and stored (4°C in the dark) pending sample preparation and analyses for nutrient concentrations and laboratory incubation experiments. Whole water samples for total phosphorus (TP) were stored in 50 mL falcon tubes at 4°C and in the dark until analysis. We also recorded the discharge (m^3^ s^–1^) using a SonTek FlowTracker handheld Acoustic Doppler Velocimeter and stream water temperature (°C) using a YSI ProODO probe.

### Analytical Methods

Whole water destined for total dissolved nitrogen (TDN) analysis were double filtered through pre-ashed 0.7 μm Whatman GF/F glass microfiber filters and 0.2 μm Isopore polycarbonate membrane filters and stored in acid washed bottles at 4 or −20°C (most samples) and in the dark until analysis. Concentrations of TDN (mg N L^–1^) were measured using the second derivative spectroscopy method following persulfate digestion (Crumpton et al., 1992). Concentrations of phosphorus in the form of TP (whole water; μg P L^–1^) were determined following persulfate digestion using the molybdate blue colorimetric assay (Murphy and Riley, 1962).

Total suspended solids (TSS) was determined gravimetrically (Rice et al., 2017) by filtering water through pre-ashed, pre-weighed 0.7 μm Whatman GF/F glass microfiber filters and drying at 60°C for 24 h. Total dry mass was determined by reweighing dried filters on a microbalance. Whole water destined for chlorophyll *a* (Chl *a*), particulate phosphorus (PP), particulate organic carbon (POC), and particulate organic nitrogen (PON) was filtered through a 60 μm mesh pre-screen. Chl *a* (μg L^–1^) was analyzed using cold ethanol extraction and fluorometric assay (ISO 10260:1992, 1992) on samples filtered through pre-ashed 0.7 μm Whatman GF/F glass microfiber filters. Chl *a* filters were frozen until analysis. PP (μg P L^–1^), POC (μg C L^–1^), and PON (μg N L^–1^) were filtered through pre-ashed 0.7 μm Whatman GF/F glass microfiber filters, dried for 24 h at 60°C, and stored at room temperature until analysis. Total PP content was measured on a separate set of filters after persulfate digestion with the molybdate blue colorimetric assay (Murphy and Riley, 1962). Total POC and PON content were measured with a CN analyzer (Vario EL III Elementar Analyzer).

### DOM Composition Measurements and Analysis

A Shimadzu TOC-VWP Total Organic Carbon Analyzer was used to measure DOC concentration (mg C L^–1^) as NPOC and DIC concentration (mg C L^–1^) as IC following sample acidification with persulfate and UV illumination. We analyzed characteristics of DOM by absorbance and fluorescence analyses. Absorbance was measured from 800 to 230 nm using a Varian Cary 50 Bio UV-Visible spectrophotometer. We calculated the specific absorbance at 254 nm (SUVA_254_; L mg^–1^ C m^–1^), which is an indicator of aromatic carbon content; larger values of SUVA_254_ indicate more aromatic DOM (Weishaar et al., 2003). Excitation emission matrices (EEMs) were generated using a Varian Cary Eclipse fluorescence spectrophotometer. Fluorescence intensities were measured at excitation wavelengths ranging from 500 to 230 nm at 5 nm increments and emission wavelengths from 600 to 270 nm at 2 nm increments. EEMs were corrected for blanks and the inner filter effect using corresponding absorbance measurements (Mobed et al., 1996; McKnight et al., 2001). The Raman peak of Milli-Q at 350 nm excitation was used as a reference value to express fluorescence intensities in Raman units (RU) (Williams et al., 2010). Based on the fluorescence measurements, four indices were calculated: (i) a spectral slope ratio used as a proxy for molecular size (S_R_; Helms et al., 2008); (ii) the β:α ratio used as an indicator of the extent of DOM degradation (Parlanti et al., 2000; Wilson and Xenopoulos, 2009); (iii) the fluorescence index used an indicator of source (FI; McKnight et al., 2001); and (iv) a humification index which reflects the extent of humification (HIX; Ohno, 2002).

Fluorescence measurements were then subjected to parallel factor (PARAFAC) analysis to reduce matrix data into discrete components. PARAFAC was conducted following the recommendation of Stedmon and Bro (2008) using the DOMFluor toolbox in MATLAB R2007b. EEMs were fit to an existing model where the results were obtained within the same catchment area (Williams et al., 2016). Prior to analysis, EEM wavelength ranges were first trimmed to 250–500 nm excitation and 300–600 nm emission. The results of the PARAFAC model revealed seven fluorescent components, three of which were identified as terrestrially sourced (C2–C4), two which were microbially derived (C5–C6), one which was protein-derived (C7), and one which appeared to have both terrestrial and autotrophic components (C1). Neither the mean fluorescence maxima (F_max_), nor the mean relative abundance of each component appeared to vary between initial and final measurements for either stream. In the agriculture stream, C1 was both the most abundant (24%) and had the highest F_max_ of about 0.89 Raman units (RU) of all the components identified by the model. C2 was the next most abundant, at 21.7% and F_max_ of 0.79 RU, followed by C6 at 19.2% and 0.70 RU and C3 at 16.3% and 0.60 RU. C5, C7, and C4 were the least abundant (about 5–7.5% each) and had the lowest F_max_ values, all around 0.20–0.30. In the forest stream, C1 was both the most abundant (around 28%) and had the highest F_max_ of about 0.86 Raman units (RU) of all the components identified by the model. C2 was the next most abundant, at 23.2% and F_max_ of 0.71 RU, followed by C3 at 18% and 0.54 RU and C6 at 13.2% and 0.40 RU. C5, C4, and C7 were the least abundant (about 5–6.5% each) and had the lowest F_max_ values, all around 0.15–0.20.

### Incubation Methods to Quantify BDOC

We conducted short term laboratory incubation experiments in order to determine DOC biodegradability. Using preliminary incubations, from stream water collected on February 18 and March 3, 2018, we determined the optimal incubation time to be of 5 days, where the majority of the DOC pool was degraded, as we aimed primarily at determining the most reactive fraction of DOC similar to Guillemette and del Giorgio (2011). We used triplicate assays for each stream on each sampling date. The incubations were run 24 times for each stream. Within 6–8 h of collection, whole water was filtered in the lab through pre-ashed 0.7 μm Whatman GF/F glass microfiber filters followed by 0.2 μm Isopore polycarbonate membrane filters, effectively removing all bacteria and microbes from the water. In addition, we prepared stream water inoculum as the source of microbes for our incubations by filtering whole water through a 60 μm mesh pre-screen. After filtration, the incubations were prepared with 90% filtered water and 10% inoculum and divided into 40 mL amber vials (acid washed and ashed). For each sample, three replicates were killed and immediately filtered through 0.2 μm Isopore polycarbonate membrane filters to serve as initial samples. An additional three replicates were loosely covered to allow excess CO_2_ produced by the degradation of DOM to escape and incubated in the dark at laboratory temperature to serve as final samples. After 5 days, the final three replicates were also filtered through 0.2 μm Isopore polycarbonate membrane filters. Samples were stored (4°C in the dark) in airtight 40 mL amber vials (acid washed and ashed) until analysis.

DOC was measured as described above. The coefficient of variation and 95% confidence interval of the DOC triplicates was very low (CV: 0–0.28 across all samples), as such the initial and final DOC triplicates were averaged before calculating BDOC (Supplementary Table A1). BDOC (% of initial DOC), or Δ DOC, was calculated as the change in DOC (lost: < 100; or gained: > 100) over the 5-day incubations (Eq. 1). Equation (1) calculates the change in DOC concentration during the incubations and assumes DOC can decrease or increase (Δ DOC).

(1) $$B\,D\,O\,C\,o\,r\,\Delta \,D\,O\,C=1-\left(\frac{D\,O\,C_{I\,n\,i\,t\,i\,a\,l}-D\,O\,C_{F\,i\,n\,a\,l}}{D\,O\,C_{I\,n\,i\,t\,i\,a\,l}}\right)\times 100$$

DOM fluorescence and absorbance analyses and PARAFAC modeling were also performed as described above. While we did not measure bacterial abundance or bacterial production as part of the incubations, bacterial abundance varies between 0.02 to 1.04 × 10^9^ cells L^–1^ (mean 0.33 × 10^9^ cells L^–1^) and bacterial production varies between 0.76 to 429 μg C L^–1^ d^–1^ (mean 88.21 μg C L^–1^ d^–1^) in these streams (Williams et al., 2010, 2012). The change in DOM is expressed as the difference between the end (i.e., final) and start (i.e., initial) value of each DOM index over the 5-day incubations and is hereafter referred to as the delta (Δ) value.

### Statistical Analyses

The Welch’s *t*-test was used to test the significant (*p* < 0.05) difference between Δ DOC in both streams, and the Pearson correlation coefficient was used to measure synchrony. Significant differences between the initial and final DOM indices between and within the streams were determined using the Wilcoxon signed-rank test. Finally, the one-sample Wilcoxon signed-rank test was used to determine whether the Δ DOM indices differed from zero in each stream (i.e., whether the DOM composition significantly changed during the incubations). All significance tests were performed in base R (R Core Team, 2018).

To further analyze the change in DOM composition in both streams, principal component analysis (PCA) was used to condense multivariate information from the initial and final DOM indices using the R package “vegan” (Oksanen et al., 2018). The PCA was calculated to visualize the variation in DOM composition in the two streams and to produce “meta-variables,” i.e., principal components, that carry the information of several measurement variables and are suitable for further use in analyses. As a result, we represented DOM composition using the principal component scores of the PC axis that best explained the variability in DOM (PC1 scores). Shifts in DOM composition were then measured using the eigenvectors of the PCA describing the initial DOM composition (based on the DOM composition of the two streams across the 24 sampling days at the start of the incubations) to predict PCA scores at the end of the incubations. The Euclidean distance was measured as the length between the initial and final points (i.e., change in DOM composition during the incubations).

Several analyses were performed to investigate the drivers of Δ DOC and the change in DOM composition during the incubations. The Spearman’s rank-order correlation coefficient was used to measure the strength and direction of association between Δ DOC and discharge, ambient water temperature, nutrient concentrations, and initial DOM composition. Statistical differences between Δ DOC and season by stream were evaluated using a two-way analysis of variance (ANOVA) with an interaction term between season and stream.

Next, general linear regressions were used to analyze the effect of discharge and ambient water temperature on the change in DOM composition during the incubations. To further analyze the effect of discharge, nutrient concentrations, and initial DOM composition on the change in DOM in both streams, we computed a canonical correlation analysis (CCA). We then measured the significance of each canonical correlation using permutations with the R package “CCP” (Menzel, 2012). Finally, we used generalized linear models to analyze the effect of discharge, ambient water temperature, and nutrient concentrations on Euclidean distance. All analyses were performed using the statistical software R (R version 3.5.1).

## Results

### Stream Conditions

Temporal patterns in discharge (*r* = 0.70; *p* < 0.001; Supplementary Figure A1A) and ambient water temperature (*r* = 0.83; *p* < 0.001; Supplementary Figure A1B) were significantly correlated and synchronous in the two streams. While ambient water temperature was significantly higher in the agriculture stream (18.42 ± 6.89°C) than in the forest stream (14.18 ± 3.82°C; Wilcoxon signed-rank test, *n* = 24, *p* < 0.001), there was no significant difference in discharge between the streams (agriculture: 0.28 ± 0.37 m^3^ s^–1^; forest: 0.27 ± 0.16 m^3^ s^–1^). Despite this, discharge varied broadly in the agriculture stream (0.0173 to 1.4959 m^3^ s^–1^, *CV* = 129.84) and less in the forest stream (0.0954 to 0.479 m^3^ s^–1^, *CV* = 43.00).

### DOC Concentration and DOM Composition

**FIGURE 1:**
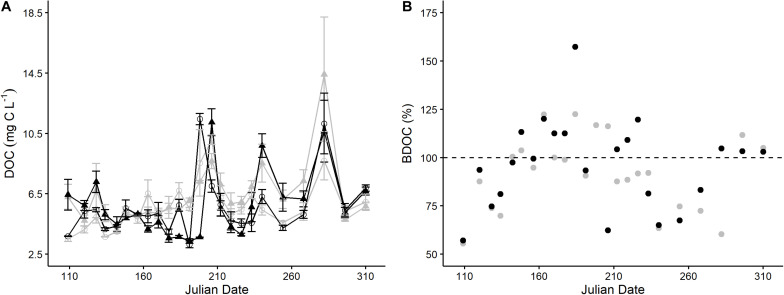
Temporal changes in DOC concentration during 5-day incubations in both streams (gray = agriculture, black = forest) **(A)**. Initial (▲) and final (∘) DOC measurements, plus standard deviation bars, are shown. Bioavailable dissolved organic carbon (BDOC) after 5-day incubations of samples collected from April to November 2018 in both streams (gray = agriculture, black = forest) **(B)**. Given as the mean proportion of change (*n* = 3 replicates), where the dashed line indicates BDOC = 100% (i.e., no change during degradation); BDOC < 100 indicates a decrease and BDOC > 100 indicates an increase in DOC concentration during the incubations.

**FIGURE 2:**
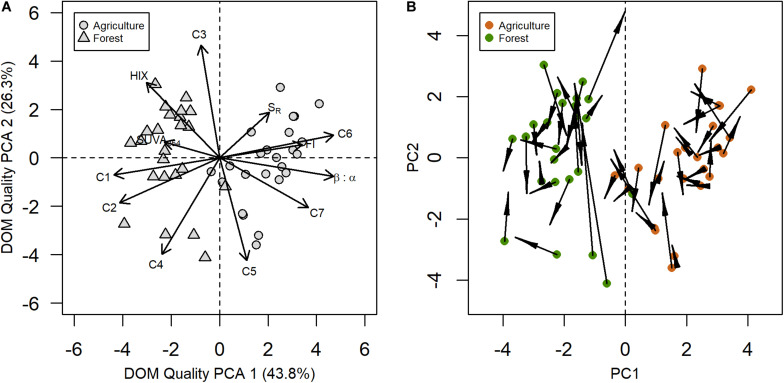
Principal component analysis (PCA) based on the initial incubation absorbance and fluorescence data from the two streams distinguishes terrestrial from microbial-like DOM **(A)**. Arrows are based on PCA structural coefficients. End coordinates are predicted scores computed with the PCA eigenvectors and the DOM composition at the end of the incubations **(B)**. Lengths of arrows denote the intensity of the shift in DOM composition.

We found that the initial and final DOC concentrations in the agriculture and forest stream incubations had similar averages and ranges (Figure 1A and Table 1). Based on our data, we did not find a significant difference in DOC concentrations between the streams (Wilcoxon signed-rank test, *N* = 24; initial: *p* = 0.06043; final: *p* = 0.7257; Figure 1A). However, there was a significant correlation between the initial DOC in the two streams (*p* = 0.02868). Alternatively, there were significant differences in the initial and final DOM composition between the streams, except S_R_ and initial C3 (Tables 1, 2). The DOM composition in the forest stream had more aromatic, humic-like properties, revealed by high SUVA_254_, HIX, and humic-like fluorescence values (Figure 2A). On the other hand, the agriculture stream had a less pronounced terrestrial signal, indicated by high β:α, FI, and comparatively higher microbial- and protein-like fluorescence (Figure 2A). This pattern was reflected by the DOM composition after the incubations as well.

**TABLE 1:** Mean ± standard deviation, minimum, and maximum values for the initial and final DOC concentrations and DOM indices measured in the laboratory incubation experiments in both streams (*n* = 24).

	**Initial**			**Final**		
	**Mean ± SD**	**Min**	**Max**	**Mean ± SD**	**Min**	**Max**
**Agriculture**						
DOC	6.40 ± 2.07	3.97	14.42	5.71 ± 1.57	3.50	10.08
S_R_	0.89 ± 0.062	0.79	0.99	0.87 ± 0.068	0.73	0.96
β:α	0.78 ± 0.032	0.71	0.84	0.79 ± 0.028	0.73	0.83
FI	1.46 ± 0.052	1.36	1.58	1.45 ± 0.054	1.36	1.60
HIX	0.91 ± 0.013	0.89	0.93	0.92 ± 0.014	0.89	0.96
SUVA_254_	2.26 ± 0.58	1.00	3.80	2.33 ± 0.42	1.50	3.13
C1	23.79 ± 1.05	21.93	26.54	24.01 ± 1.11	22.33	27.01
C2	21.23 ± 1.01	19.52	22.98	21.73 ± 1.01	20.09	23.40
C3	16.83 ± 2.04	11.32	21.16	16.34 ± 1.53	13.81	19.30
C4	5.17 ± 1.16	2.76	7.35	5.10 ± 0.94	2.79	6.98
C5	7.46 ± 1.00	5.54	9.53	7.60 ± 0.93	6.10	9.06
C6	18.77 ± 1.77	15.15	21.38	19.18 ± 1.82	16.45	23.06
C7	6.75 ± 0.90	4.90	8.12	6.05 ± 0.96	4.11	7.67
**Forest**						
DOC	5.72 ± 2.15	3.37	11.24	5.58 ± 1.99	3.15	11.45
S_R_	0.88 ± 0.022	0.84	0.93	0.88 ± 0.025	0.83	0.93
β:α	0.66 ± 0.038	0.59	0.76	0.66 ± 0.026	0.59	0.70
FI	1.36 ± 0.039	1.29	1.46	1.37 ± 0.041	1.31	1.45
HIX	0.93 ± 0.016	0.90	0.97	0.94 ± 0.013	0.91	0.96
SUVA_254_	2.65 ± 0.56	1.70	3.50	2.67 ± 0.56	1.07	3.42
C1	27.60 ± 1.72	25.57	32.24	28.09 ± 1.76	25.70	32.97
C2	23.24 ± 0.73	21.71	25.07	23.20 ± 1.02	19.72	24.34
C3	18.01 ± 3.35	9.72	22.47	17.99 ± 2.49	13.52	25.16
C4	6.11 ± 1.21	3.91	8.74	6.04 ± 1.13	3.88	8.10
C5	6.58 ± 1.07	5.24	9.96	6.51 ± 0.93	3.42	8.16
C6	13.01 ± 1.61	9.99	15.28	13.19 ± 1.92	9.13	17.10
C7	5.46 ± 0.87	4.07	7.27	4.99 ± 0.77	3.66	6.62

*Variable units are as follows: DOC (mg C L^–1^), SUVA_254_ (L mg^–1^ C m^–1^), and components C1–C7 (% F_max_).*

**TABLE 2:** Wilcoxon signed-rank tests between the agriculture and forest stream incubations tested separately for their initial and final DOC concentrations and DOM indices (*n* = 24).

	**Initial**	**Final**
DOC	ns	ns
S_R_	ns	ns
β:α	<0.001	<0.001
FI	<0.001	<0.001
HIX	<0.001	<0.001
SUVA_254_	<0.001	<0.001
C1	<0.001	<0.001
C2	<0.001	<0.001
C3	ns	<0.01
C4	<0.01	<0.001
C5	<0.01	<0.001
C6	<0.001	<0.001
C7	<0.001	<0.001

*Results indicate that DOM composition largely differed between the streams at the start and conclusion of the incubations.*

### DOC Degradation and Production

The portion of bioavailable DOC (BDOC) did not significantly differ between streams (agriculture: 91.77% ± 19.38%; forest: 96.42% ± 23.03%). BDOC and Δ DOC values ranged from 55.59 to 122.53% and from 57.11 to 157.43% in the agriculture and forest streams, respectively, and were synchronous throughout the sampling period (*r* = 0.61, *p* < 0.01; Figure 1B). Nine agriculture and 11 forest stream incubations resulted in Δ DOC values over 100% (i.e., DOC concentrations increased during the incubations) (Figure 1B). In addition, BDOC and Δ DOC values were similarly dispersed in the agriculture (*CV* = 21.12) and forest streams (*CV* = 23.88). Despite their differing initial DOM compositions, the streams experienced simultaneous fluctuations in their biodegradability and production, and the degradation of carbon during the incubations was the same in both streams.

### Changes in DOM Composition During Incubations

**FIGURE 3:**
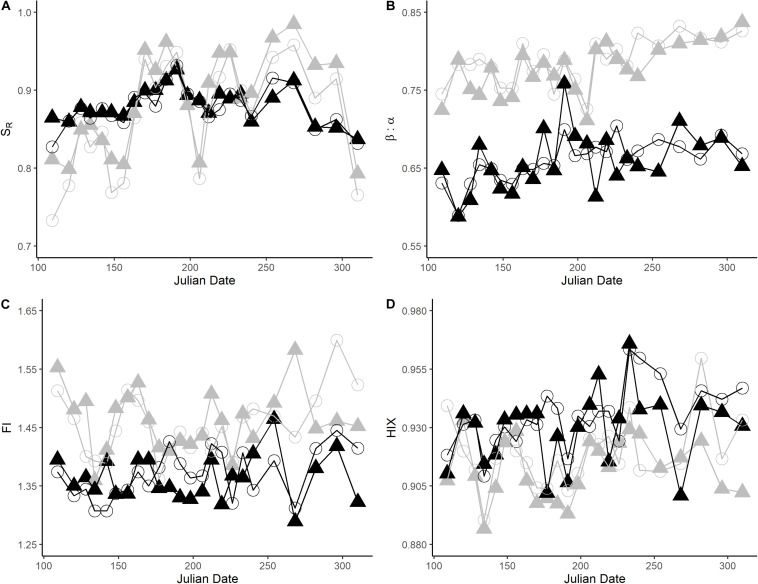
Temporal changes in four selected DOM indices during 5-day incubations: **(A)** slope ratio (S_R_), **(B)** freshness index (β:α), **(C)** fluorescence index (FI), and **(D)** humification index (HIX) in both streams (gray = agriculture, black = forest). Initial (▲) and final (∘) DOM measurements are shown. Additional DOM indices are available in the Supplementary Information.

During the incubations we saw increases in DOM indices that resulted in both an enhanced allochthonous- and autochthonous-like DOM signature, particularly in the agriculture stream, as indicated by increases in HIX and components C1 and C2, and increases in β:α and component C6, respectively (Figure 3 and Supplementary Figure A2). We also saw a decrease in DOM molecular size in ∼70% of incubations, as determined by lower S_R_ values, in both streams (Figure 3A). The decrease in molecular weight was especially pronounced in the agriculture stream (75% of time sampled) compared to the forest stream (∼60%). DOM composition related to fresher, more autochthonous-like material also shifted during the incubations as indicated by upwards trends in β:α in both streams, as well as FI in the forest stream (Figures 3B,C). In particular, incubations resulted in fresher material as measured using β:α in 70 and 55% of incubations in the agriculture and forest streams, respectively. Greater humification, as measured using HIX, was also found after incubation, especially in the agriculture stream on October 9, 2018 (Figure 3D). When temporal values are averaged, β:α (*p* < 0.05), HIX (*p* < 0.05), C1 (*p* < 0.001), C2 (*p* < 0.001), and C6 (*p* < 0.05) increased, while S_R_ (*p* < 0.001) and C7 (*p* < 0.001) decreased, in the agriculture stream (Table 1). Similarly, there was an increase in C1 (*p* < 0.001) and decrease in C7 (*p* < 0.05) in the forest stream incubations.

**FIGURE 4:**
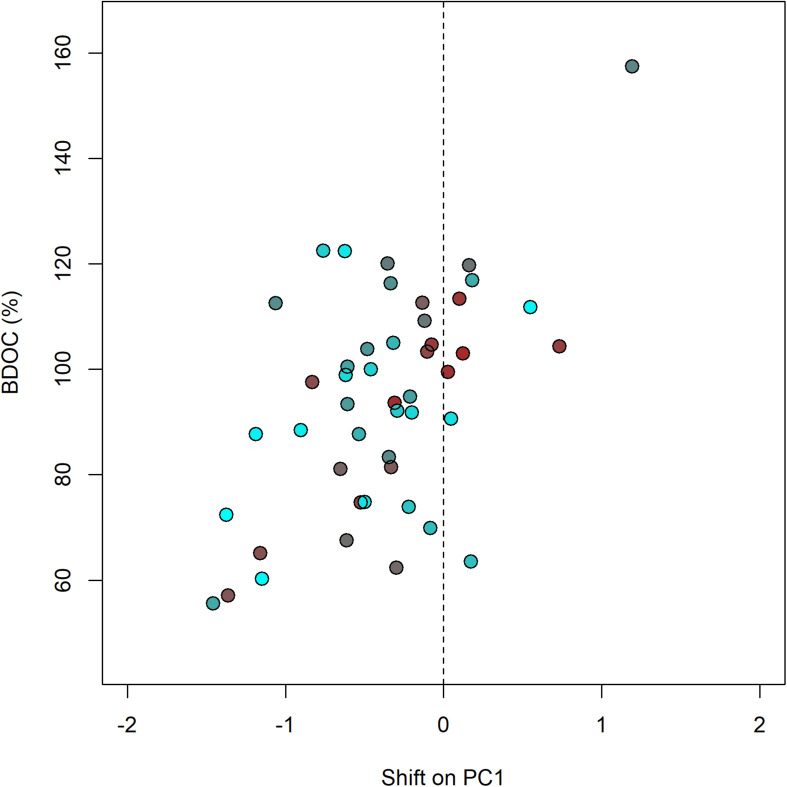
BDOC increases with the absolute shift in DOM composition along PC1 (i.e., each stream’s final PC1 scores minus the respective initial score before degradation) (*r* = 0.42; *p* < 0.01). Initial DOM composition as derived from Figure 2A (PC1) is indicated by color. Brown color denotes stream samples with predominantly terrestrial DOM and blue color represents streams with relatively more autochthonous-like DOM.

We further depicted shifts in DOM composition occurring during the incubations by using the eigenvectors of the PCA describing the initial DOM composition to predict PCA scores at the end of the incubations (Figure 2B). This revealed that there was no identifiable pattern to how the DOM composition changed during the incubations for either stream. On average, the PC1 scores (from Figure 2A) decreased in both streams during the incubations. This either means that the initial terrestrial imprint on DOM increased upon microbial degradation or the relative proportion of allochthonous-like fluorescence increased as autochthonous-like fluorescence was utilized, which resulted in an enhanced allochthonous-like DOM signature. However, this trend was not consistent across samples, with increases in both humic- and microbial-like DOM indices during the incubations in both streams. We also investigated the Euclidean distance between the initial and final points (i.e., change in DOM composition during the incubations) and found that there was a significant difference between the agriculture and forest streams (*p* < 0.05). Overall, the forest stream exhibited a significantly greater change in DOM composition during the incubations than the agriculture stream. Finally, relating the length of these shifts in DOM composition to Δ DOC, we found that greater shifts in DOM composition during the incubations induced higher values and a greater change in DOC (*r* = 0.42; *p* < 0.01; Figure 4).

### Linking DOM Composition and Seasonality to DOC Degradation and Production

The two study streams drain catchments covered by mainly agriculture or forest, respectively, which results in differences in nutrient concentrations and DOM composition. However, these differences in catchment land use and stream water chemistry were not reflected in the change in DOC concentration or DOM composition in the streams. We found few significant correlations with Δ DOC. Chl *a* (*r* = 0.45; *p* < 0.05) and initial SUVA_254_ (*r* = 0.73; *p* < 0.001) both had a positive relationship with Δ DOC in the agriculture stream. Meanwhile, TDN (*r* = 0.55; *p* < 0.01), initial SUVA_254_ (*r* = 0.65; *p* < 0.001), and initial C1 (*r* = −0.50; *p* < 0.05) were all significantly correlated with Δ DOC in the forest stream. Overall, Δ DOC increased with elevated Chl *a* (agriculture), TDN (forest), and SUVA_254_ values (both), and decreased with higher proportions of component C1 (forest). However, the initial PC1 scores were not significantly correlated with Δ DOC (*p* = 0.3917), and we did not find a relationship between the initial DOM composition and the change in DOC during the incubations. Additionally, despite fluctuations in the degradation and production of DOC throughout the year, neither discharge nor ambient water temperature were correlated with Δ DOC in either stream.

A two-way ANOVA was conducted to compare Δ DOC values in the spring, summer, and autumn seasons in both streams. None of the variables had a significant effect on Δ DOC [interaction: *F*_(2,41)_ = 0.024, *p* = 0.98; season: *F*_(2,41)_ = 3.13, *p* = 0.054; stream: *F*_(1,41)_ = 0.671, *p* = 0.42]. Next, the initial and final DOM indices were plotted for each stream and color-coded by discharge to determine whether seasonality may be an important factor regulating the degradation of DOM during the incubations. While it appeared as though discharge could be an important predictor for some DOM indices, there were no significant relationships between ambient water temperature and Δ DOM. Linear regressions of Δ DOM with discharge as a predictor were significant for some variables: Δ S_R_ (*r* = 0.16; *p* < 0.05) in the agriculture stream, and Δ SUVA_254_ (*r* = 0.14; *p* < 0.05), Δ C2 (*r* = 0.19; *p* < 0.05), Δ C3 (*r* = 0.16; *p* < 0.05), and Δ C5 (*r* = 0.22; *p* < 0.05) in the forest stream. Overall, Δ S_R_ (agriculture) and Δ C3 (forest) decreased with increasing discharge levels, while Δ SUVA_254_, Δ C2, and Δ C5 all increased in with higher discharge in the forest stream, indicating that higher discharge in the forest stream during sample collection resulted in an enhanced humic-like DOM composition at the end of the incubations.

**FIGURE 5:**
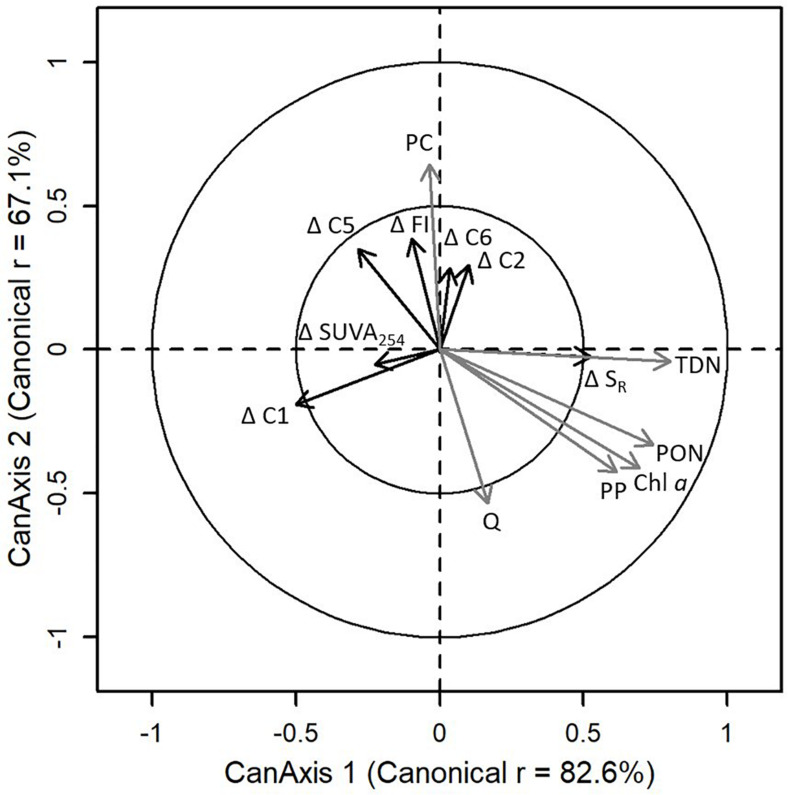
Canonical correlation analysis (CCA) based on discharge (Q), nutrient concentrations, initial DOM composition (PC scores; where higher values indicate a more allochthonous-like DOM composition), and Δ DOM indices in both streams (where gray = predictor variables; and black = response variables). Pillai’s trace from F-distribution was significant (*p* < 0.001).

We also used CCA to further evaluate the relationship between discharge, nutrient concentrations, and initial DOM composition (PC scores; where higher values indicate a more allochthonous-like DOM composition) with the change (Δ) in DOM composition during the incubations in both streams (Figure 5). Low nutrient concentrations coincide with increased changes in DOM composition during the incubations. Additionally, we found a positive correlation between initial DOM composition (PC) and many Δ DOM indices, including Δ FI, Δ C2, Δ C5, and Δ C6. Increased allochthonous DOM contributions led to a greater change in DOM composition during the incubations.

Finally, we investigated the shifts in the DOM pool (using Euclidean distances from Figure 2B) to further explore the effects of discharge, ambient water temperature, and nutrient concentrations. There was a significant relationship between discharge and Euclidean distance in the agriculture stream (*p* < 0.001; r-squared = 0.39). We also found significant relationships between several nutrients and Euclidean distance in the agriculture stream: TSS (*p* < 0.01; r-squared = 0.30), POC (*p* < 0.001; r-squared = 0.41), PON (*p* < 0.05; r-squared = 0.15), PP (*p* < 0.001; r-squared = 0.40), DIC (*p* < 0.01; r-squared = 0.32), TDN (*p* < 0.05; r-squared = 0.14), and TP (*p* < 0.05; r-squared = 0.18). There was a greater change to the complete DOM pool in the agriculture stream incubations when samples were taken during higher discharge and when nutrient concentrations were higher (except DIC).

## Discussion

In freshwater ecosystems, the microbial degradation of DOM is expected to vary due to differences in its composition. In our study, we did not detect an enhanced biodegradability of DOC in the agriculture stream despite it being characterized by an autochthonous-like, presumably labile DOM signature. We did, however, see a greater shift in DOM composition during incubations when the initial DOM composition was more terrestrial allochthonous-like. This observation further challenges the traditional idea that DOM of terrestrial origin is less accessible and more recalcitrant to microbial activity (McCallister and del Giorgio, 2012; Fasching et al., 2014; Marín-Spiotta et al., 2014; Stadler et al., 2020). Our findings potentially contribute to the changing view that DOM composition, as measured in our study, does not necessarily predict its bioavailability.

### DOC Degradation and Production

Δ DOC ranged from 55.6 to 157.4% in both streams. Previous studies looking at biodegradability in streams did not report any BDOC measurements exceeding 100%, while we found that about 40% of our incubations had values over 100%, an indication of organic matter production. When OM production was detected, the average increase in DOC was ∼0.7 mg L^–1^ for both streams. This small increase could simply be attributed to differential C moieties sampled by our TOC Analyzer using the wet oxidation technique (Yoon et al., 2018). It is also possible that the methods we used to carry out the incubations resulted in those Δ DOC values exceeding 100%: using native bacteria to inoculate the incubations, not supplementing the stream water with nutrients, or only letting the bioassays incubate for 5 days which may not have been long enough to quantify DOC degradation during specific times of the year when the DOM pool was more recalcitrant. However, the low coefficient of variation between replicates (<0.28, mean = 0.08) and high degree of temporal synchrony between the two study streams gives us confidence in the Δ DOC results. While we did not measure bacterial abundance or bacterial production in this study, we hypothesize that the increasing DOC concentrations during the incubations could possibly be a return of organic carbon to the DOC pool either because of viral lysis (viral shunt) of bacterial cells (Middelboe and Lyck, 2002; Weinbauer, 2004; Boras et al., 2009) or because of new microbial production and biomass (Kaiser and Benner, 2008). The conditions in the bioassays may have supported a rapid increase in microbial activity and the subsequent release of dissolved organic material into the DOM pool from cell lysis (viral or otherwise; Teira et al., 2003; Kawasaki et al., 2013). The amount of bacterial inoculum likely also varied through time (Koetsier et al., 1997) thus likely explaining the temporal variability in Δ DOC that we found. Finally, these streams harbor a functionally diverse microbial community that has been shown to vary with DOM composition (Fasching et al., 2020). Together, our results point to the importance of bacterial contribution to the DOM pool, at least for part of the time series and for short 5-day incubation periods. Contributions of bacterially derived carbon to DOC pools have been documented to be in the range of 30–50% (Kawasaki et al., 2013). Nevertheless, our results should be interpreted with caution given that DOC production was unexpected here.

BDOC values in most studies range from 0 to 70% (Fellman et al., 2009; Fasching et al., 2014; Coble et al., 2016, 2019). Variation in microbial biodegradability and production may be due to differences in laboratory incubation setup. Coble et al. (2019) quantified BDOC using 7-day laboratory incubations, which is most like our bioassay experiments, while other studies conducted their incubations for 20–30 days (Fellman et al., 2009; Fasching et al., 2014; Coble et al., 2016). Most incubations, including ours, took place at around 20°C, however, Fellman et al. (2009) incubated their samples at 25°C. Most importantly, even though every study used a bacterial or microbial inoculum, each inoculum was created differently. Similar our study, Coble et al. (2016) used an inoculum that was site specific. However, they prepared an inoculum that introduced a microbial assemblage from the water column and from the benthos (Coble et al., 2016), while we only utilized the bacterial community in the water column. Some studies used a composite, whether it was a slurry made up of river sediment, riparian soil, and river water (Coble et al., 2019) or a combination prepared by leaching soil collected in the riparian zone and incubating before using (Fellman et al., 2009).

Previous studies have found that changes to the DOM pool are related to differing catchment land uses, nutrient loading, and rates of microbial carbon processing (Wilson and Xenopoulos, 2009; Williams et al., 2010). Because of this, we expected to find that the bioavailability of DOC in streams increases with greater agricultural land use and a higher amount and proportion of autochthonous-like DOM. For example, Asmala et al. (2013) found that a higher percentage of forests and peatlands in the catchment decreased the bioavailability of DOC. However, we found that Δ DOC was not significantly different in the two streams. In fact, Δ DOC was correlated between our study streams, suggesting that the temporal patterns of Δ DOC were synchronous (Coble et al., 2016). Intriguingly, our data also suggest that different compositions of DOM and microbial populations can have similar biodegradability and production depending on the time of year. This temporal synchrony is likely driven by physical and climatic factors experienced by all streams and rivers in the region, such as changes in discharge, ambient water temperature, or light availability. Because our bioassays were incubated in the lab under similar conditions it is more likely that the synchrony is driven by biological successions such as microbial community composition, and its interactions among environmental factors (Coble et al., 2016; Vogt et al., 2016; Stadler et al., 2020).

### Changes in DOM Composition During Incubations

Although we saw a greater change in the DOM composition in the forest stream, there were significant changes in DOM composition in both streams during the incubations. Besides the quantitative changes (as indicated by DOC concentration), DOM composition shifted upon microbial degradation, resulting in a slightly enhanced humic-like composition in both streams, similar to Boyd and Osburn (2004). Increases in humic-like fluorescence can be expected if microbial humification processes dominated, with bacteria selectively degrading the bioavailable DOM and, in the process, generating humic-like fluorescence (Guillemette and del Giorgio, 2012; Shimotori et al., 2012). Also, it may be possible that DOM typically considered to be allochthonous-like can be produced by microbial activity, potentially resulting from degradation of protein-like components (Guillemette and del Giorgio, 2012; Stadler et al., 2020). With increasing values of both allochthonous and autochthonous DOM indices, there was no discernible pattern across stream type or time though. Finally, we found that greater shifts in DOM composition during the incubations also increased the change in DOC.

### Linking DOM Composition and Seasonality to DOC Degradation and Production

We did not find a relationship between Δ DOC and the initial DOM composition, so DOM composition was not a good predictor for Δ DOC in our study. While the DOM indices we commonly measure are good tracers, they do not represent the complete DOM pool. Therefore, the fact that we did not find a relationship may indicate that there is no effect of DOM composition on Δ DOC, and further studies are needed to support this hypothesis. The lack of significant relationships could also suggest limited predictive ability of Δ DOC by DOM composition measured here, similar to other studies (Fasching et al., 2014; Coble et al., 2016; Williams et al., 2016). This means that the initial qualities of DOM determined by catchment characteristics had no detectable impact on defining the biodegradability or production of DOC, which is consistent with Stadler et al. (2020) and refutes our main hypothesis. In contrast, other studies reported that the DOM source (i.e., different catchment land uses) significantly affected and was an influential predictor of DOC degradation (Fellman et al., 2009; Wickland et al., 2012), specifically that the amount of forests in the catchment decreases the bioavailability of DOC (Boyer and Groffman, 1996; Asmala et al., 2013).

Allochthonous-like DOM is typically more aromatic and complex and thus considered to have less biodegradable compounds (Kaplan and Bott, 1989; Kalbitz et al., 2003a; Berggren et al., 2009) but not always (e.g., McCallister and del Giorgio, 2012). Aged fractions of allochthonous-like DOM may be preferentially consumed, stimulating bacterial metabolism (McCallister and del Giorgio, 2012). Streams with more colored, aromatic terrestrial-derived DOM have higher ecosystem respiration than those dominated by protein-like DOM (Catalán et al., 2018) a result consistent with our findings here. In addition to DOM composition, microbial production also depends on the availability of other organic and inorganic forms of nitrogen and phosphorus (Brookshire et al., 2005; Harbott and Grace, 2005). Thus, further research is needed to measure a wider array of variables that are known to change or may possibly change the size of the bioavailable DOM pool.

Shifts in land use from forest cover to agriculture not only alter the chemical complexity of DOM, but it often also reduces the riparian zone along agricultural streams, further impacting DOM composition and bioavailability (Giling et al., 2013). Our comparable Δ DOC measurements could also be because the incubations were carried out exclusively in the dark, thus inhibiting photodegradation. The tree canopy cover above the agriculture stream is more open than above the forest stream, allowing higher light availability and consequently allowing for more a photobleached initial DOM pool due to photodegradation. Nevertheless, despite the streams differing in land use, nutrient concentrations, and DOM composition, the BDOC pool was similar, indicating the importance of a yet unmeasured or unknown variable.

We did find that Δ DOC and initial SUVA_254_ were positively correlated in both streams, similar to Coble et al. (2019). This increase in SUVA_254_ values could indicate either the selective utilization of less aromatic DOM by microbes or the production of UV-absorbing compounds by bacteria (Ortega-Retuerta et al., 2009). Aromatic compounds are also likely to be the most stable and dominant fraction of DOM (Kalbitz et al., 2003b). Finally, aromatic DOM can also play an important consumptive role in metabolism by acting as an electron shuttle in redox reactions (Cory and McKnight, 2005).

Although we did not find a significant link to BDOC in our study, there was a relationship between Δ DOM and both initial DOM composition and nutrient concentrations. There was a greater shift in DOM composition during incubations when the initial DOM composition was more terrestrial allochthonous-like. This is consistent with brown-water streams in Austria (Fasching et al., 2014). This may indicate that DOM of terrestrial origin is substantially reworked once it enters aquatic ecosystems to meet the microbial energy demand (Fasching et al., 2014), especially if low concentrations of nutrients are present. This also agrees with the greater shift in DOM composition that we saw in the forest stream incubations. Similarly, Fasching et al. (2014) did not find a relationship between DOM composition and BDOC but found that DOM composition impacted microbial carbon use efficiency (a determinant of primary production). Although we did not find a relationship with BDOC in our study, microbial respiration (e.g., DIC or CO_2_) may still be impacted by DOM composition. This would mean that a similar amount of DOM could be taken up by microbes in both streams (depending on seasonal factors) but degraded and reworked in different ways with possible consequences for respiration and CO_2_ production.

Finally, the bioavailability of DOM has previously been reported to have strong seasonal variation (Lønborg et al., 2009; Sintes et al., 2010). We did see strong fluctuations throughout the year and no correlation between either discharge or ambient water temperature and BDOC. However, our linear models showed that increases in discharge in the forest stream were related to shifts in DOM that lead to a more humic-like composition. Meanwhile, increases in discharge and nutrient concentrations lead to an increase in the change in DOM composition in the agriculture stream. In general, there was no relationship between BDOC and season, which is surprising considering BDOC fluctuated simultaneously in the two streams and suggests that perhaps the changing microbial community or some other climatic control that we did not measure is affecting BDOC.

## Conclusion

We found very few significant relationships between DOC biodegradability and production, and DOM composition, discharge, and ambient water temperature. The synchronous pattern of Δ DOC cannot be explained by DOM composition or seasonality alone, suggesting that broad environmental factors also shape Δ DOC. However, the initial DOM composition is important for the reworking of the DOM pool. The lack of discernible pattern in DOM change (i.e., increases in both allochthonous- and autochthonous-like DOM indices) during the incubations also needs to be further explored. When small parts of the DOM pool are transformed in aquatic ecosystems there can be significant impacts on ecosystem function, such as decreases in primary productivity, releasing of CO_2_ to the atmosphere, or burial of carbon in sediments (Prairie, 2008; Lapierre et al., 2013; Seekell et al., 2015). Now that we are re-evaluating the common assumption that terrestrial DOM is resistant to microbial activity, we need to continue to investigate the metabolic fate of terrestrial DOM and assess its potential contribution to carbon cycling in streams.

## Data Availability Statement

All datasets generated for this study are included in the article/Supplementary Material, further inquiries can be directed to the corresponding author.

## Author Contributions

MK, CF, and MX designed the experiment. MK collected the data, conducted the experiment, and wrote the first draft. MK and CF analyzed the results. MX coordinated the study. All authors contributed to the article and approved the submitted version.

## Conflict of Interest

The authors declare that the research was conducted in the absence of any commercial or financial relationships that could be construed as a potential conflict of interest.
