# Supplementary material for: Synchronous Biodegradability and Production of Dissolved Organic Matter in Two Streams of Varying Land Use
Source: Front Microbiol. 2020 Nov 16;11:568629. doi: 10.3389/fmicb.2020.568629 (PMC7701103; doi:10.3389/fmicb.2020.568629)
Supplement: Supplementary file 1 [file Data_Sheet_1.docx]

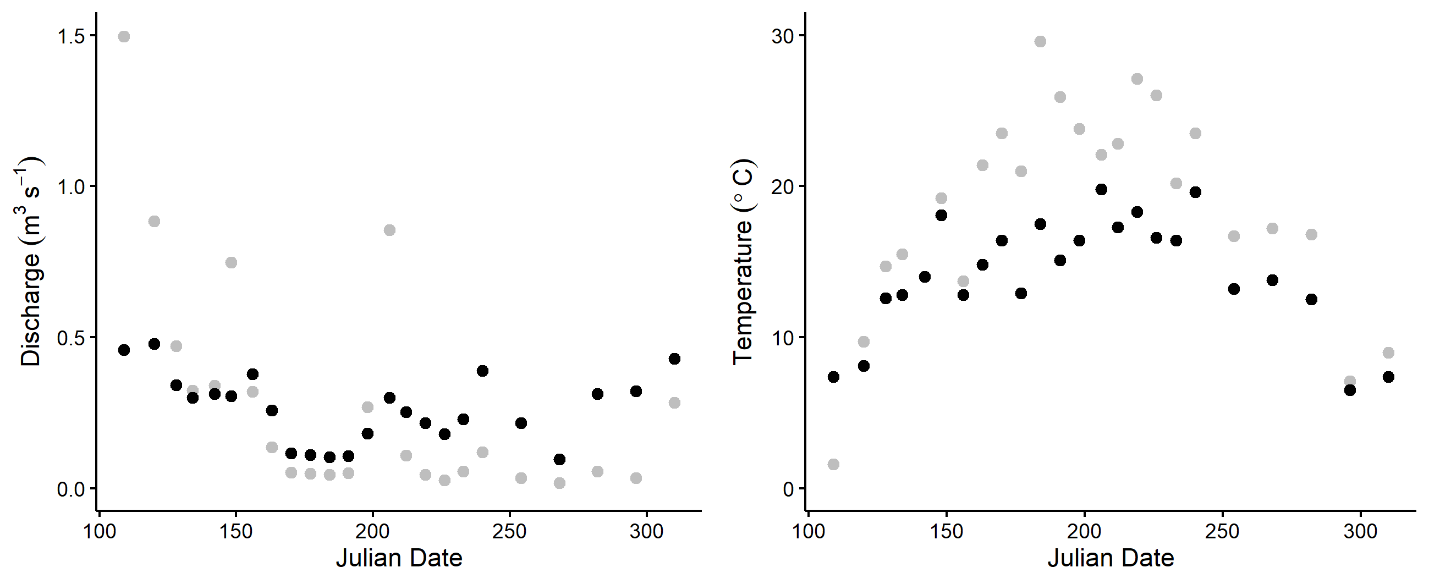


**Figure A1**. Discharge (A) and ambient water temperature (B) measured during sampling in both streams from April to November 2018 (grey = agriculture, black = forest).


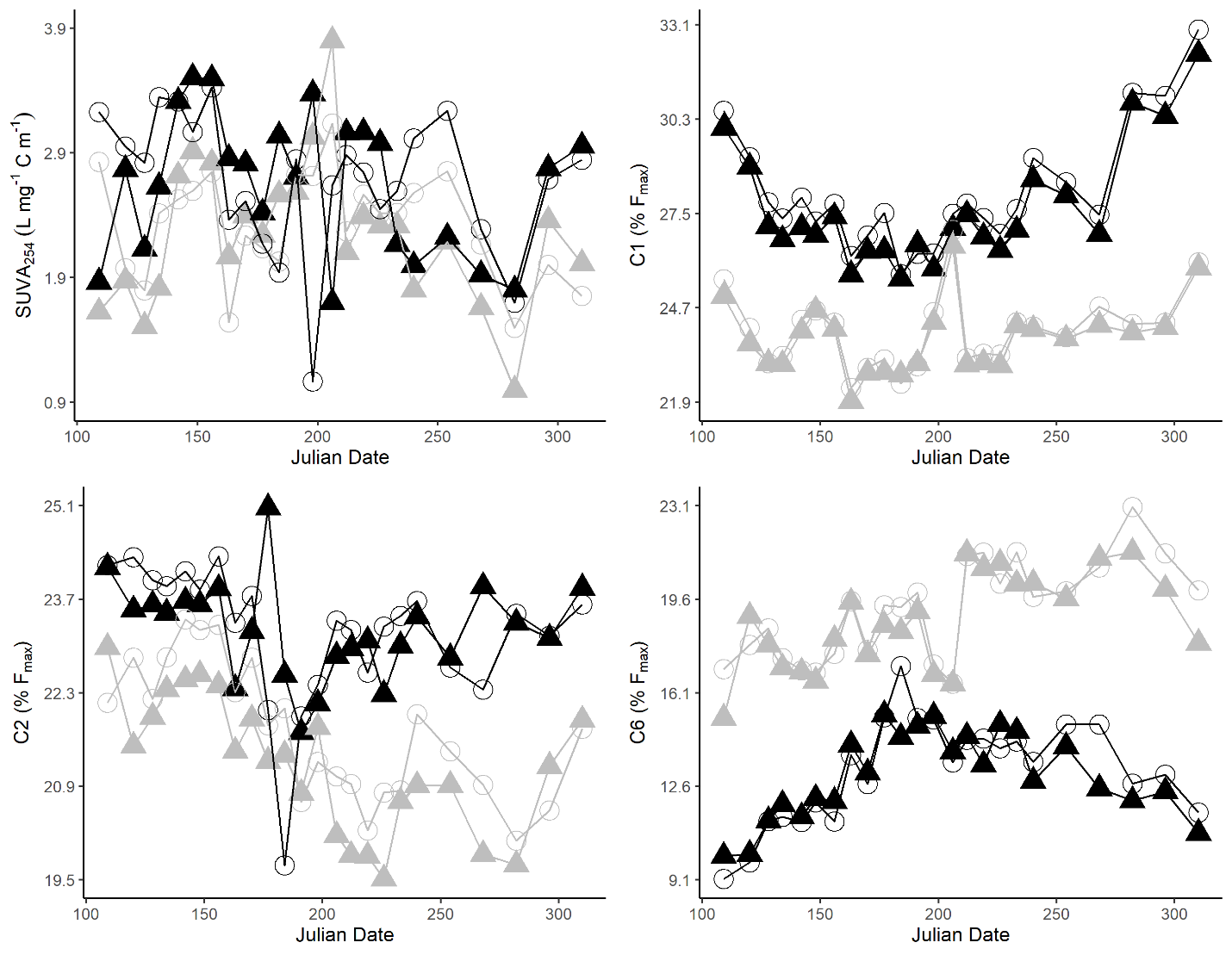


**Figure A2**. Temporal changes in four selected DOM indices and PARAFAC components during 5-day incubations: (A) SUVA_254_, (B) C1, (C) C2, and (D) C6 in both streams (grey = agriculture, black = forest). Initial (▲) and final (○) DOM measurements are shown.

**Table A1**. Mean ± 95% confidence interval (n = 3 replicates) for the initial and final DOC concentrations (mg C L^-1^) measured in the laboratory incubation experiments from April to November 2018 in both streams.

|  | **Agriculture** | | **Forest** | |
| --- | --- | --- | --- | --- |
| **Julian Date** | **Initial** | **Final** | **Initial** | **Final** |
| 109 | 6.30 ± 1.62 | 3.50 ± 0.31 | 6.44 ± 2.00 | 3.68 ± 0.05 |
| 120 | 4.73 ± 0.71 | 4.15 ± 0.47 | 5.73 ± 0.64 | 5.37 ± 0.93 |
| 128 | 6.72 ± 3.54 | 4.97 ± 0.81 | 7.29 ± 1.38 | 5.45 ± 0.27 |
| 134 | 5.22 ± 1.10 | 3.65 ± 0.03 | 5.12 ± 0.67 | 4.15 ± 0.17 |
| 142 | 3.97 ± 0.04 | 4.00 ± 0.09 | 4.48 ± 0.95 | 4.37 ± 0.16 |
| 148 | 5.12 ± 0.28 | 5.32 ± 0.62 | 4.88 ± 0.21 | 5.54 ± 1.10 |
| 156 | 5.06 ± 0.21 | 4.80 ± 0.50 | 5.15 ± 0.04 | 5.13 ± 0.18 |
| 163 | 5.32 ± 1.92 | 6.52 ± 1.78 | 4.17 ± 0.41 | 5.01 ± 0.83 |
| 170 | 5.20 ± 0.68 | 5.20 ± 0.43 | 4.63 ± 0.78 | 5.21 ± 1.42 |
| 177 | 5.59 ± 1.44 | 5.53 ± 0.65 | 3.55 ± 0.51 | 4.00 ± 0.32 |
| 184 | 5.48 ± 0.22 | 6.71 ± 1.04 | 3.64 ± 0.19 | 5.74 ± 0.76 |
| 191 | 6.06 ± 0.31 | 5.50 ± 0.19 | 3.37 ± 0.29 | 3.15 ± 0.44 |
| 198 | 7.31 ± 0.14 | 8.55 ± 4.33 | *** | *** |
| 206 | 8.67 ± 0.97 | 10.08 ± 0.62 | 11.24 ± 1.76 | 7.01 ± 0.80 |
| 212 | 7.13 ± 1.45 | 6.25 ± 1.55 | 5.79 ± 0.99 | 6.25 ± 1.48 |
| 219 | 5.86 ± 1.39 | 5.19 ± 0.63 | 4.31 ± 0.76 | 4.71 ± 1.17 |
| 226 | 5.96 ± 0.70 | 5.47 ± 0.62 | 3.80 ± 0.15 | 4.55 ± 0.56 |
| 233 | 6.96 ± 0.79 | 6.41 ± 0.46 | 5.62 ± 1.89 | 4.58 ± 1.14 |
| 240 | 8.53 ± 2.44 | 5.42 ± 0.65 | 9.69 ± 1.53 | 6.32 ± 0.93 |
| 254 | 6.13 ± 1.19 | 4.59 ± 0.22 | 6.28 ± 1.81 | 4.24 ± 0.34 |
| 268 | 7.35 ± 1.47 | 5.33 ± 1.14 | 6.16 ± 1.03 | 5.13 ± 0.51 |
| 282 | 14.42 ± 7.42 | 8.70 ± 2.51 | 10.65 ± 4.02 | 11.15 ± 3.94 |
| 296 | 4.78 ± 0.15 | 5.34 ± 0.72 | 5.27 ± 0.67 | 5.44 ± 0.82 |
| 310 | 5.67 ± 0.53 | 5.95 ± 1.00 | 6.68 ± 0.54 | 6.89 ± 0.41 |
